# Epidemiological characteristics and influencing factors of fatal drowning in children under 5 years old in Hunan Province, China: case-control study

**DOI:** 10.1186/s12889-019-7241-z

**Published:** 2019-07-17

**Authors:** Zhiyu Liu, Fanjuan Kong, Lei Yin, Aihua Wang, Lili Xiong, Donghua Xie, Lizhang Chen, Xiaoqi Sheng

**Affiliations:** 1 Information Management Division, Hunan Provincial Maternal and Child Health Care Hospital, Changsha, Hunan Province China; 2 Hunan Provincial Center for Disease Control and Prevention, Changsha, Hunan Province China; 3 Department of Epidemiology and Health Statistics, Xiangya School of Public Health, Central South University, Changsha, Hunan Province China; 4 Hunan Provincial Maternal and Child Health Care Hospital, Changsha, Hunan Province China

**Keywords:** Fatal drowning, Epidemiological features, Influencing factors, Children under 5 years old

## Abstract

**Background:**

Drowning is still the primary cause of death in children under 5 years old, however, little is known about the drowning of Hunan province children. This study identifies the previously unpublished incidence and characteristics of fatal drowning in children of Hunan Province, and provide a basis for formulating strategies for children’s survival, development and protection.

**Methods:**

Data were collected through sampling with the multistage stratified cluster. The case group included all fatal frowning children under 5 years old in 13 districts between October 2015 and September 2016. The control group was matched 1:1.The epidemic features and influencing factors of fatal drowning were analyzed retrospectively according to descriptive analysis, conditional univariate and multivariate logistic regression analysis.

**Results:**

For children aged 0–4 years, the fatal drowning was 16.1/100000 in Hunan Province. Drowning rates were higher for boys than girls. The proportion of rural areas is much higher than that of urban areas. The 1–2 years age-group was the highest of all age groups. Fatal drowning mainly occurred in summer. The three leading drowning locations were pond, ditch and well. Playing close to the water were the leading activities that preceded fatal drowning. Multivariate logistic regression analysis showed that: children with primary caregiver education in high school and above (OR = 0.05) have a lower risk of fatal drowning; children with full-time care (OR = 0.17) have a lower risk; children who received unintentional drowning safety education (OR = 0.23) have a lower risk of fatal drowning. Children who were always swimming or playing near the water in the past 6 months (OR = 3.13) have a higher risk of fatal drowning.

**Conclusion:**

The fatal drowning among children under 5 years is the result of the interaction of multiple factors. A significant number of child deaths could have been prevented if parents and other close relatives had been more concerned about the safety of their children. We should develop health education plans for villagers to warn them of the dangers of drowning and preventive measures.

## Background

Drowning is the process of experiencing respiratory impairment from submersion/immersion in liquid [1]. Respiratory injury is followed by breath-holding and involuntary laryngospasm that leads to hypercapnia, hypoxemia, and, if prolonged, respiratory or cardiorespiratory arrest [2, 3].

Globally, drowning is still the leading cause of death among children aged 1–4 years worldwide [4]. Drowning is also one of the top three causes of death among Chinese teenagers and children [5]. According to WHO estimates, about 112,000 people die from unintentional drowning in China each year [6]. In Hunan Province, the mortality rate of children under 5 years decreased from 20.4 per thousand in 2002 to 5.6 per thousand in 2016,but in the past five years, drowning has been the leading cause of death for children under 5 years old. This is an important factor endangering the health and lives of children in this region [7, 8].

The intervention of children’s drowning is urgent. However, most of the research on fatal drowning in World and China is based on current research, and there are few case-control studies. Hunan Province also lacks relevant reports.

Therefore, this study intends to use a 1:1 paired case-control study to understand the epidemiological characteristics and related influencing factors of fatal drowning in children under 5 years old in Hunan Province. The risk factors of drowning death of children under 5 years old in Hunan Province were discussed. To supplement and perfect the shortcomings of descriptive research, and this paper puts forward the intervention strategies and measures of child drowning in our province.

## Methods

### Research object

Hunan Province is located in southeastern China, with an area of 21.2 km^2^ and a population of 69.0 million, there are 123 counties (cities, districts) and 14 cities. The province is a water-oriented province with long and hot summers. There are 5341 rivers more than 5 km, over 14,000 reservoirs, and numerous ponds in the province. Multistage stratified cluster sampling was adopted. The first stage was stratified according to the city, and the second stage was stratified according to the district. In the 11 cities, 13counties (cities, districts) of Hunan Province, all unintentional drowning cases with children under the age of 5 from October 1, 2015 to September 30, 2016 were collected from the Maternal and Child Health Information Direct Reporting Management System. According to the 1:1 matching method, children under 5 years old who had never experienced unintentional drowning in the previous year before the survey were selected as controls. The matching conditions were as follows: (1) The age difference between the case and control was within 6 months; (2) The control was the same gender as case; (3) The control was living in the same village (residential) committee or adjacent village (residential) committee; (4) The control was a healthy child without illness. When the control child met the above conditions, the child was included.

### Survey method

The survey was conducted in four quarters. For each quarter, the staff of each district organized and reported the drowning death list of children under 5 years old through the network direct reporting system. The list is checked with the CDC, civil affairs, public security and other departments, and checked by spot checks at the provincial, municipal and county levels to ensure that the death list is accurate and reliable. Using a retrospective questionnaire survey, in the two quarters after the child’s drowning, trained investigators were accompanied by a child care professional and the local village doctors to investigate the case and the primary caregiver of the control child. The questionnaire consisted of two parts. The first part was shared by the Children’s Drowning Death Questionnaire and the Control Questionnaire, and included basic child condition, basic family condition and child care. The second part was unique to the Children’s Drowning Death Questionnaire, which investigated the situation when drowning occurred.

### Statistical methods

EpiData3.1 was used to build related databases, and data entry was done using double entry. Statistical analyses were performed using SPSS20.0. The main characteristics were described by the adoption rate and proportion of counting data, and the influencing factors were analyzed by logistic regression under the conditions of single factor (first =0.10) and multiple factors (first =0.10, second =0.15). The Cox regression model in the survival analysis was used to fit and calculate the corresponding *P* value, OR value and OR value 90% CI.

## Results

### General characteristics

From October 1st, 2015 to September 30th, 2016, 85 cases of fatal drowning occurred in children under 5 years old in 13 counties (cities, districts) of Hunan Province. A total of 79 cases were investigated in the study. 6 cases were not investigated because the caregivers did not cooperate or went out to work. The survey rate was 92.9% (79/85). During the investigation period, the prevalence of fatal drowning in children under 5 years in Hunan Province was 16.1/100,000(Table 1).

**Table 1:** Incidence of fatal drowning in children under 5 years old in Hunan Province

Counties (cities, districts)	Under 5 years Old child	The number of drowning (investigation)	The number of drowning (actual)	Incidence rate(1/100,000)
Liuyang	97,958	6	6	6.1
Hetang	13,696	2	2	14.6
Lusong	13,940	5	5	35.9
Shifeng	15,963	1	2	12.5
Xiangxiang	53,213	18	18	33.8
Leiyang	64,706	12	14	21.6
Pingjiang	63,702	9	9	14.1
Anxiang	21,694	6	6	27.7
Anhua	64,673	3	4	6.2
Guiyang	44,046	8	8	18.2
Xintian	23,109	1	3	13.0
Zhijiang	22,108	4	4	18.1
Yongshun	30,314	4	4	13.2
Total	529,122	79	85	16.1

### Epidemiological characteristics

During the investigation period (Table 2), a total of 51 boys and 28 girls fatally drowned. The majority of drowning events occurred in toddlers aged 1–2 years who made up 43.0%. The lowest proportion was in the 0–1 year who made up 5.1%. Summer showed consistently higher frequency patterns for all drowning deaths (40.51%).July was the month that drowning events most commonly occurred(19.0%), but he number of occurrences in January was the least(3.8%). Most fatal drownings (93.7%) occurred in rural areas.

**Table 2:** Epidemic features of fatal drowning in children under 5 years old in Hunan Province

Epidemic features	Number of cases (person)	Composition ratio (%)
Total	79	
Gender		
Male	51	64.6
Female	28	35. 4
Age range (years)		
0~	4	5.1
1~	34	43.0
2~	18	22.8
3~	13	16.5
4~	10	12.7
Season		
Spring	17	21.5
Summer	32	40.5
Autumn	17	21.5
Winter	13	16.5
Address		
Urban area	2	2.5
Suburbs	3	3.8
Rural	74	93.7
Water type village		
Open pit	5	6.3
Reservoir	7	8.9
Pond	37	46.8
Ditch	10	12.7
Water tank/bucket	4	5.1
Bathtub	2	2.5
Well	8	10.1
Rivers/Lakes	3	3.8
Dam	1	1.3
Brook	1	1.3
Sewer	1	1.3
Main activities before the occurrence of drowning		
Take a bath	1	1.3
Plying in the water	4	5.1
Playing by the water	70	88.6
Slip when wading	3	3.8
Falling from the upstairs	1	1.3
Place of death		
Where the drowning occurred	63	79.8
Medical institution	11	13.9
On the way to the hospital	5	6.3
Primary caretaker		
Parents	33	41.8
Grandparents and uncles	43	54.4
Neighbors	3	3.8

The top five fatal water types were ponds, ditches, wells, reservoirs and open pits. The most important activity of children before the occurrence of fatal drowning was playing close to the water, followed by playing in the water and slipping when wading.One case occurred in the bath, and one case fell from the second floor of the house that was opened to the pit. Most of the children’s death sites were in the places where the drowning occurred, accounting for 79.8% of the total number of occurrences. The rest were in medical institutions and during medical treatment.

Univariate conditional logistic regression analysis was performed on 24 influencing factors (Table 3), and possible influencing factors were selected and included in the multivariate conditional logistic regression model (Table 4). The results suggest that the primary caregivers were highly educated, children had full-time care, and children had received unintentional water safety education, which was a protective factor for fatal drowning in children under 5 years. Consistently swimming or playing near the water for the previous 6 months was a risk factor for fatal drowning in children under 5 years .

**Table 3:** Table of assignments of variables

Variable name	Assignment situation
Case control	0 = Control
	1 = Case
the relationship between caretaker and children	0 = Father/mother
	1 = Grandparent
Education level of primary caregivers	1 = Elementary school and below
	2 = Junior high school
	3 = High school and above
Smoking status of primary caregivers	1 = Often
	2 = Occasionally
	3 = Never
Drinking situation of primary caregivers	1 = Often
	2 = Occasionally
	3 = Never
physical condition of primary caregivers	0 = Health
	1 = Sick
Are there other children in the family?	0 = No 1 = Yes
Is it a left-behind child?	0 = No 1 = Yes
Total annual income of the family	1 = < 20,000 2 = 20,000~79,999 3 = ≥80,000
Is there a water storage container in the house?	0 = No 1 = Yes
Whether the container holds water	1 = Often
	2 = Occasionally
	3 = Never
Whether the container has a lid or fence	0 = No 1 = Yes
Is there a water body near the place of residence?	0 = No 1 = Yes
Whether the water body has a lid or fence	0 = No 1 = Yes
Is there a medical institution within 500 m of the current residence?	0 = No 1 = Yes
Does the child have full-time care?	0 = No 1 = Yes
Father’s time spent with children in the last month	1 = <7 h 2 = 7~20 h
	3 = 21~34 h 4 = ≥35 h
Mother’s time spent with children in the last month	1 = <7 h 2 = 7~20 h
	3 = 21~34 h 4 = ≥35 h
The most common way to take a bath for children in the past month	0 = bathtub
	1 = take a shower
Caregiver swimming ability	1 = can not swim
	2 = not very skilled
	3 = skilled
Caregivers think that children should be taught to swim	0 = No 1 = Yes
Has the caregiver taught children to swim?	0 = No 1 = Yes
Have children ever swum or played by the water in the past 6 months?	1 = Often
	2 = Occasionally
	3 = Never
Whether the caregiver has the emergency rescue knowledge of drowning	0 = No 1 = Yes
Has the child(≥1 year) received safety education for unintentional drowning?	0 = No 1 = Yes

**Table 4:** Multi-factor conditional logistic regression analysis of fatal drowning in children under 5 years old in Hunan Province

Influencing factor	β	*S*_*b*_	Wald*χ*^2^	*P*	*OR*	90%*CI*(%)
Education level of primary caregivers						
Elementary school and below					1.00	
Junior high school	−1.58	0.61	6.75	< 0.01	0.21	0.08–0.56
High school and above	−3.07	1.07	8.28	< 0.01	0.05	0.01–0.27
Does the child have full-time care?						
No					1.00	
Yes	−1.75	0.68	6.71	0.01	0.17	0.06–0.53
Have children ever swimming or playing by the water in the past 6 months?						
Never					1.00	
Occasionally	0.98	1.05	0.88	0.35	2.67	0.48–15.02
Often	1.14	0.54	4.49	0.03	3.13	1.29–7.60
Has the child (≥1 year)received safety education for unintentional drowning?						
No					1.00	
Yes	−1.46	0.70	4.32	0.04	0.23	0.07–0.74

## Discussion

This study found that the prevalence of fatal drowning in children under 5 years old was 16.1/100,000 in Hunan Province, which was lower than previous studies in Hunan Province [9]. But it was higher than the prevalence of fatal drowning in children under 5 years in Guangdong Province (10.1/100,000) [10]. The prevalence of fatal drowning was also higher than the children and adolescents in China determined through meta-analysis (8.98/100,000) [11]. This was because drowning usually occurs in early childhood [12]. A population-based cross-sectional study of 32 OECD countries found that:Drowning is a high priority public health problem in Eastern Europe,central Asia,Japan and the USA [13].The government and society should provide a strategic direction and framework for guiding multisectoral actions and evaluate related work.

Males has a greater risk of drowning than females at any location [14], but particularly in pools [15]. This study showed that boys are especially at risk of drowning, nearly with twice the overall mortality rate of girls. It’s similar to many other studies [16–18]. Studies have reported that boys are more active and daring [19]. It may be that boys and girls differ in their personality, behavior, cognition and parents educate boys and girls differently about safety.

The majority of drowning events occurred in toddlers aged 0–4 years, who made up 76 and 72% of Australian indigenous and non-indigenous drowning incidents, respectively [20].In China, children in the 1–4 years group are most prone to drowning [21]. This study showed that for drowning, the proportion of children aged 1 to 2 is the highest, which is consistent with the results of a survey in Jiangsu Province [22]. A possible reason is that children aged 1 to 2 have just learned to walk. They are curious about the surrounding environment and underestimate the danger, and their scope of activity is gradually increased but they lack self-protection abilities. Fatal drowning in children under 5 years in Hunan Province is high in summer, which is the same as in most studies [20, 23]. It may be because the summer has high temperatures and abundant rainfall. Children are easily separated from the caretaker and go to the water to play or swim. The proportion of drowning deaths among children in rural areas exceeded 90%, which is consistent with the results of previous surveys in our province [24]. A summary of the drowning incidence in low- and middle-income countries found that of the 4932 cases of drowning, 84% had died in rural areas with most deaths in Southeast Asian countries [25]. Generally, the low economic levels in rural areas lead to backward infrastructure, unregulated daily management, low education levels of parents, insufficient awareness of drowning, and guardians that are not careful enough to look after the child.

Many countries were found to be failing to report sufficiently codes in drowning mortality data submitted to the WHO [26], which makes it nearly impossible to compare locations and influencing factors between countries. This study shows that the top five water bodies of drowning are ponds, ditches, wells, reservoirs, and open pits. Only a few water bodies have fences or warning signs. Although the occurrence of children’s drowning in Guangxi Province [27], Jiangxi Province [28], Jiangsu Province [22] and other places are not the same, drowning cases are mainly concentrated in natural, open waters. In high-income countries, most children’s drowning occurs in swimming pools, so installing fences or barriers with automatic closures and self-locking doors is an effective prevention strategy [29]. However, swimming pools are less common in low-income countries, especially in rural areas [19]. In low- and middle-income countries, observational evidence and studies on drowning indicate that natural waters are the greatest risk of drowning in children and that covering open waters or installing fences may be impractical, especially in areas with many nearby waters [30]. Raising public awareness, strengthening health education, strengthening supervision, installing door barriers at the door of the residence, using playpens, or using a fence to designate a safe play area at home or while out is an easy and affordable alternative and a helpful monitor [31–33].

The most prominent activity before fatal drowning in children under 5 years in Hunan Province was playing close to the water. Most of the sudden deaths of children are caused by unintentional exposure to water. In this study, 79.8% of the drowning cases died at the scene, and 6.3% died on the way to medical treatment. In Suzhou [34], more than half of children under the age of 5 years cannot receive timely treatment after drowning. In Haimen [35], 90.9% of child fatalities from drowning occurred in their homes or on their way to hospital. The reason for this phenomenon is as follows: first, the caretaker’s poor supervision means they cannot find the children that have fallen into the water in time; second, the rural areas are underdeveloped, so it is difficult to get drowning children to the hospital in time; and third, many people do not have the skills to perform cardiopulmonary resuscitation. Although rescue and recovery have a limited impact on reducing drowning mortality and incidence, in the event of drowning, rescue and recovery can determine whether a person lives or dies [36].

Multivariate conditional logistic regression analysis indicated that a high level of education of the primary caregiver was a protective factor for the occurrence of fatal drowning in children under 5 years in Hunan Province. Some studies [37] note that the cultural level of caregivers affects the occurrence of drowning in rural children and that children in families with lower education levels are at greater risk of fatal drowning. The survey found that the main caregivers of children under 5 years are grandparents who have low levels of education, lack safety awareness, common sense and knowledge about drowning, also poorly supervise children. Full-time nursery care is a protective factor for the occurrence of fatal drowning in children under 5 years. A number of investigations have shown that children are prone to drowning accidents when caregivers neglect them [25, 38]. Unintentional drowning safety education for children is a protective factor for fatal drowning in children under 5 years of age. Some studies [39] have indicated that caregivers sometimes/often inform children that safety knowledge is a factor in drowning protection. The main caregivers should strengthen their safety education regarding children’s contact with water, especially in outdoor waters. Swimming or playing near the water in the past 6 months was a risk factor for fatal drowning, which was similar to other studies [38, 40]. Children like to play with water; their cognitive functional development is not perfect, and they lack sufficient awareness of the dangerous environment. Therefore, when they play, swim or wash things at the water’s edge, they are prone to falling into the water, leading to death. The exposure level of a dangerous environment is an important factor in determining whether sudden death can occur [41].

However, there are still some limitations in this study. Firstly, the survey questionnaires involved individuals, family privacy and some situations when drowning occurred. Some children caregivers were at work or refused an investigation, or were unwilling to answer the sensitive questions, which may affect the representativeness of the sample. Secondly, consider that the family members’ emotions and acceptance, local customs, organizational coordination, etc. There was a time interval between the time of the questionnaire and the occurrence of drowning, and it was difficult to avoid recall bias of the respondents during the investigation. Third, the disadvantage of cluster sampling is that the sampling error caused by cluster sampling is often larger than that of simple random sampling because of the great difference between different groups. Based on the above limitations, the scope of the research, the survey area, the credibility of respondents and the extrapolation of the results can be expanded in future research.

## Conclusion

Drowning is an important cause of injury-related death, also in Hunan province. Fatal drowning incidence of children under 5 years in Hunan province is presented for the first time. The quality of data from this study identifies risk factors and gives good insight for interventions to target specific sociodemographic, age and gender groups. Rural areas are the main areas of drowning. Ponds, ditches and wells dominate the drowning burden for under 5 years and produced the most fatalities. Most drowning occurred because of playing near the water. Therefore, more communities should implement day care to ensure continued adult supervision, especially during the day. In addition, small ponds and irrigation ditches should be fenced or drained, and ponds/Wells should be covered with grates to prevent children from falling into them. Finally, all of the risk factors of drowning should be taken into account when designing preventive measures and family education. These preventative strategies are especially important in rural areas where the risks of drowning are higher.

## Data Availability

The datasets used and/or analysed during the current study are available from the corresponding author on reasonable request.
